# Characteristics and clinical evaluation of X chromosome translocations

**DOI:** 10.1186/s13039-023-00669-7

**Published:** 2023-12-21

**Authors:** Ning Huang, Jihui Zhou, Wan Lu, Laipeng Luo, Huizhen Yuan, Lu Pan, Shujun Ding, Bicheng Yang, Yanqiu Liu

**Affiliations:** 1 Medical Genetics Center, Jiangxi Maternal and Child Health Hospital, Nanchang, 330006 China; 2 Maternal and Child Health Hospital of Nanchang Medical College, Nanchang, 330006 China; 3 Medical Laboratory, Jiangxi Maternal and Child Health Hospital, Nanchang, 330006 China

**Keywords:** Reciprocal translocation, X chromosome, Karyotyping, Genetic counselling

## Abstract

**Background:**

Individuals with X chromosomal translocations, variable phenotypes, and a high risk of live birth defects are of interest for scientific study. These characteristics are related to differential breakpoints and various types of chromosomal abnormalities. To investigate the effects of X chromosome translocation on clinical phenotype, a retrospective analysis of clinical data for patients with X chromosome translocation was conducted. Karyotype analysis plus endocrine evaluation was utilized for all the patients. Additional semen analysis and Y chromosome microdeletions were assessed in male patients.

**Results:**

X chromosome translocations were detected in ten cases, including seven females and three males. Infantile uterus and no ovaries were detected in case 1 (FSH: 114 IU/L, LH: 30.90 mIU/mL, E2: < 5.00 pg/ml), and the karyotype was confirmed as 46,X,t(X;22)(q25;q11.2) in case 1. Infantile uterus and small ovaries were both visible in two cases (FSH: 34.80 IU/L, LH: 17.06 mIU/mL, E2: 15.37 pg/ml in case 2; FISH: 6.60 IU/L, LH: 1.69 mIU/mL, E2: 23.70 pg/ml in case 3). The karyotype was detected as 46,X,t(X;8)(q13;q11.2) in case 2 and 46,X,der(X)t(X;5)(q21;q31) in case 3. Normal reproductive hormone levels and fertility abilities were found for cases 4, 6 and 7. The karyotype were detected as 46,X,t(X;5)(p22.3;q22) in case 4 and 46,X,der(X)t(X;Y)(p22.3;q11.2) in cases 6 and 7. These patients exhibited unremarkable clinical manifestations but experienced a history of abnormal chromosomal pregnancy. Normal phenotype and a complex reciprocal translocation as 46,X,t(X;14;4)(q24;q22;q33) were observed in case 5 with a history of spontaneous abortions. In the three male patients, multiple semen analyses confirmed the absence of sperm. Y chromosome microdeletion and hormonal analyses were normal. The karyotypes were detected as 46,Y,t(X;8)(q26;q22), 46,Y,t(X;1)(q26;q23), 46,Y,t(X;3)(q26;p24), respectively.

**Conclusions:**

Our study provides insights into individuals with X chromosome translocations. The clinical phenotypes are variable and unpredictable due to differences in breakpoints and X chromosome inactivation (XCI) patterns. Our results suggest that physicians should focus on the characteristics of the X chromosome translocations and provide personalized clinical evaluations in genetic counselling.

## Background

Reciprocal translocations are common chromosomal abnormalities that are reported to occur in approximately 0.2% of humans and 2.1% of couples who experience spontaneous abortion [1, 2]. X chromosome translocations are extremely rare and are estimated to occur in 1:30,000 live births [3]. With balanced chromosomal structural rearrangements of no gain or loss of genetic material, the individuals are expected to be phenotypically normal but at a high risk for infertility, spontaneous abortion, embryo failure, and birth defects due to cytogenetically unbalanced pregnancies. This imbalance consists of partial monosomies and/or trisomies that may affect the chromosomal segments involved in the translocations [4, 5]. In contrast to autosomal translocations, X chromosome translocations are quite rare structural abnormalities of chromosomes. The clinical phenotype associated with X chromosome translocation is variable. In practice, certain individuals exhibit unremarkable clinical manifestations. Patients with X-autosome translocations are likely to experience short stature, mental retardation, germinal aplasia and X-linked disorders due to X chromosome inactivation [6–8]. With X–Y translocations females are phenotypically normal or short stature according to the breakpoint of the X chromosome, and anomalies of gonadal development tend to occur in males [9]. Additionally, X chromosome translocation patients have a higher rate of offspring with X chromosome aneuploidy than patients with autosomal translocation for X chromosome inactivation [10, 11]. This study aimed to investigate laboratory data and phenotypes in X chromosome translocations to provide meaningful clinical evidence for genetic counselling and reproductive risk assessment.

## Results

Between January 2021 and February 2023, 30,815 patients visited the Medical Genetics Center of Jiangxi Maternal and Child Health Hospital for clinical genetic consultation. Ethical approval was obtained from the Ethics Committee of Jiangxi Maternal and Child Health Hospital. Among the total study population (N = 30,815), females (N = 16,543) including infertility, history of spontaneous abortion, history of abnormal pregnancy, amenorrhea, and males (N = 14,272) including infertility underwent karyotyping. Of these, ten cases of X chromosome translocations were detected. In cases 1, 2 and 3, secondary amenorrhea, infantile or small uterus, and absent or small ovaries were observed. In cases 4 and 6, an abnormal chromosomal pregnancy was shown. In cases 5 and 7, there was a spontaneous abortion history. Cases 8, 9, and 10 involved three men with infertility due to azoospermia (Table 1).

**Table 1:** General information of the 10 cases

Case	Gender	Age	Clinical manifestation
1	Female	16	secondary amenorrhea, secondary sex characteristics normal, infantile uterus, size 24 × 20 × 14 mm, uterine cavity linear, no obvious endometrium, and both ovaries were not detectable
2	Female	21	secondary amenorrhea, infantile uterus, size 32 × 25 × 20 mm, uterine cavity linear, left ovary likely size 19 × 15 × 12 mm, right ovary size 25 × 17 × 14 mm
3	Female	25	preprimary ovarian insufficiency, secondary amenorrhea, oral oestrogen sequential therapy, infertility for 1 year, uterus size 40 × 34 × 29 mm, endometrium 8 mm, left ovary likely size 19 × 20 × 16 mm, right ovary size 25 × 15 × 16 mm
4	Female	36	B ultrasonography indicated the foetus had tetralogy of Fallot, intense left heart spot, single umbilical artery and small kidneys, prenatal diagnosis from the amniotic fluid cells suggested that the karyotype of the foetus was 47,XN, +der(X)t(X;5)(p22.3;q32)mat
5	Female	34	early spontaneous abortion twice
6	Female	31	B ultrasonography indicated the foetus had short limbs, NIPT revealed sex chromosome abnormality, prenatal diagnosis from cells in the amniotic fluid suggested that the karyotype of the foetus was 46,Y,der(X)t(X;Y)(p22.3;q11.2)mat
7	Female	38	during first marriage a healthy girl was born, induced abortion 1 time and spontaneous abortion 1 time, infertility for 1 year during remarriage, uterus size 52 × 45 × 40 mm, endometrium 5 mm, left ovary size 26 × 20 × 16 mm, right ovary size 31 × 21 × 18 mm
8	Male	24	azoospermia
9	Male	32	azoospermia
10	Male	45	azoospermia

X chromosome translocations were detected in 10 cases. Patients 1, 2 and 3 with amenorrhea underwent B-ultrasound scanning. The reference average size is 7 × 4 × 2 cm for uterus, 4 × 3 × 1 cm for the ovaries. Patients 4 and 6 with a history of abnormal chromosomal pregnancy underwent amniocentesis and karyotyping of the foetus

**Fig. 1:**
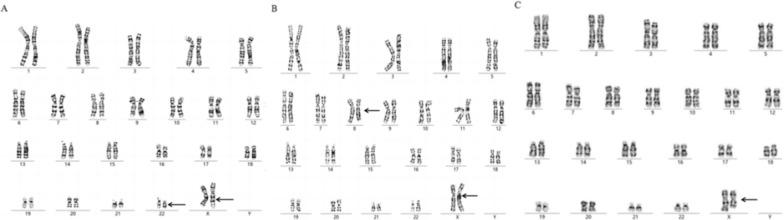
**A** The karyotype was 46,X,t(X;22)(q25;q11.2) for case 1. **B** The karyotype was 46,X,t(X;8)(q13;q11.2) for case 2. **C** The karyotype was 46,X,der(X)t(X;5)(q21;q31) for case 3

Anti-Müllerian hormone (AMH) < 0.01 ng/ml was observed in cases 1, 2 and 3 with secondary amenorrhea. Transabdominal ultrasound revealed an infantile uterus and small ovaries in cases 2 and 3 (Follicle-stimulating hormon (FSH): 34.80 IU/L, Luteinizing hormone (LH): 17.06 mIU/mL, Oestradiol (E2): 15.37 pg/ml; FISH: 6.60 IU/L, LH: 1.69 mIU/mL, and E2: 23.70 pg/ml) compared to no ovaries in case 1 (FSH: 114 IU/L, LH: 30.90 mIU/mL, E2: < 5.00 pg/ml). A significant correlation was observed between AMH levels and ovarian insufficiency. The karyotypes were detected as 46,X,t(X;22)(q25;q11.2) for case 1, 46,X,t(X;8)(q13;q11.2) for case 2, and 46,X,der(X)t(X;5)(q21;q31) for case 3 (Fig. 1). According to karyotype, the breakpoints located on the long arm of the X chromosome (Xq25 for case 1, Xq13 for case 2, and Xq21 for case 3) correlated with low AMH levels and ovarian insufficiency. Balanced reciprocal translocations between X chromosome and autosomes were observed in cases 1 and 2, and imbalanced translocation was observed in case 3.

**Fig. 2:**
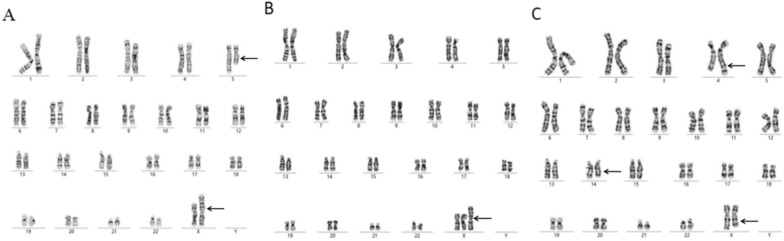
**A** The karyotype was 46,X,t(X;5)(p22.3;q22) for case 4. **B** The karyotype was 47,XN, +der(X)t(X;5)(p22.3;q32)mat for the foetus of case 4. **C** The karyotype was 46,X,t(X;14;4)(q24;q22;q33) for case 5

**Fig. 3:**
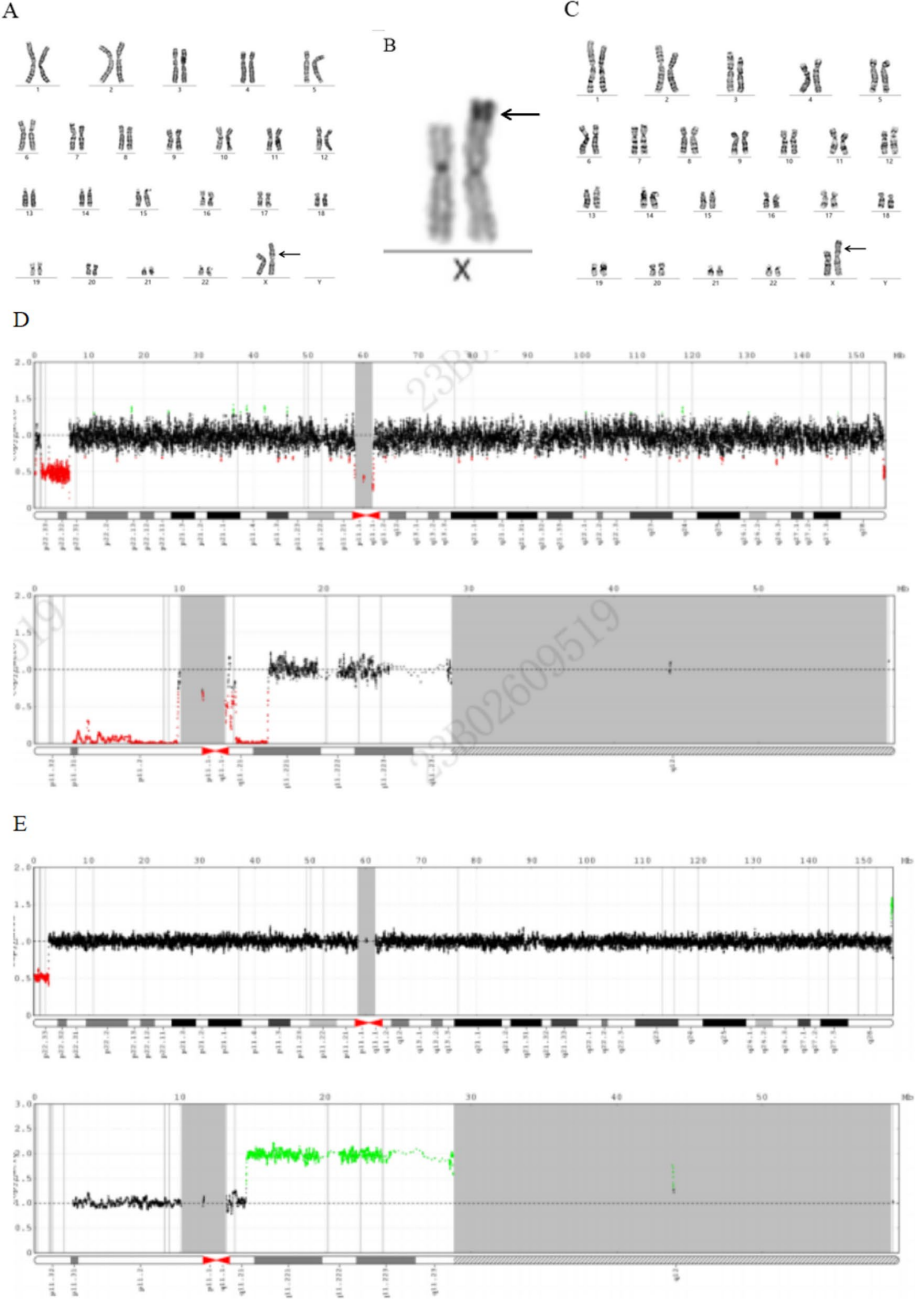
**A** The karyotype was 46,X,der(X)t(X;Y)(p22.3;q11.2) for case 6. **B** Heterochromatin visualized and ligated to Xp22.3 in the C-band for case 6. **C** The karyotype was 46,X,der(X)t(X;Y)(p22.3;q11.2) for case 7. **D** CNV-seq revealed seq[GRCh37]Xp22.33p22.31(990,745–6426956) × 1 chrX:g.990745-6426956del chrY: 16,207,792–28819361 for case 6. **E** CNV-seq revealed seq[GRCh37]del(X)(p22.33p22.33) chrX:g.287328-2757513del seq[GRCh37]dup(Y)(q11.21q12) chrY:g.14567885-28819361dup for the foetus of case 7

Additionally, Cases 4 and 6 involved normal hormonal levels and fertility abilitiy but with a history of abnormal chromosomal pregnancy. The karyotypes were 46,X,t(X;5)(p22.3;q22) and 46,X,der(X)t(X;Y)(p22.3;q11.2). In case 4, ultrasonography at 20 weeks of gestation indicated that the foetus had tetralogy of Fallot, an intense left heart spot, a single umbilical artery, and small kidneys. Prenatal diagnosis from the amniotic fluid cells suggested that the karyotype of the foetus was 47,XN, +der(X)t(X;5)(p22.3;q32)mat with duplication of Xp22.3-qter and 5q32-qter as confirmed by chromosomal microarray (Fig. 2). The 5q35-qter duplication syndrome presents a phenotype of mental and growth retardation, microcephaly, cardiac defects, pulmonary hypertension, genital defects, brachydactyly, congenital hipdysplasia, dental caries and eczema. The foetus in case 4 also exhibited abdominal, limb, and cardiac malformations. Imbalanced translocations between X and Y were present in cases 6 and 7. The G-banding karyotype was established as 46,X,der(X)t(X;Y)(p22.3;q11.2) and C-banding karyotype revealed heterochromatin ligated to Xp22.3 (Fig. 3). In case 6, karyotype and copy number variation sequencing (CNV-seq) of amniofluid cells revealed 46,Y,der(X)t(X;Y)mat for the male foetus inheriting der(X) from the mother. The foetus had short limbs due to SHOX haploinsufficiency associated with Leri-Weill dyschondrosteosis (LWD). In case 5 which involved spontaneous abortion 2 times, normal hormone levels were detected and complex reciprocal translocation among chromosomes X, 14, and 4 was identified by karyotyping (Table 2).

**Table 2:** Endocrine evaluation of females with X chromosome translocations

Case	Karyotype	AMH	FSH	LH	E2	PROG	PRL	TESTO
		(ng/ml)	(IU/L)	)mIU/mL)	(pg/ml)	(ng/ml)	(ng/ml)	(ng/dl)
1	46,X,t(X;22)(q25;q11.2)	< 0.01	114	30.90	< 5.00	< 0.05	24.60	7.90
2	46,X,t(X;8)(q13;q11.2)	0.01	34.80	17.06	15.37	< 0.10	9.41	25.78
3	46,X,der(X)t(X;5)(q21;q31)	< 0.01	6.60	1.69	23.70	0.68	20.0	73.90
4	46,X,t(X;5)(p22.3;q22)	2.44	3.74	10.50	106	2.64	23.6	37.90
5	46,X,t(X;14;4)(q24;q22;q33)	2.35	7.07	5.81	15.90	0.27	20.6	37.30
6	46,X,der(X)t(X;Y)(p22.3;q11.2)	1.39	6.26	4.27	32.90	0.20	11.4	< 2.50
7	46,X,der(X)t(X;Y)(p22.3;q11.2)	1.77	3.92	3.20	32.70	0.43	19.0	52.0

Seven females with X chromosome translocations underwent endocrine evaluation

AMH reference range: 1.22–11.7 ng/ml for 20–24 years, 0.890–9.85 ng/ml for 25–29 years, 0.576–8.13 ng/ml for 30–34 years, 0.147–7.49 ng/ml for 35–39 years. FSH reference range: 3.5–12.5 IU/L for the follicular phase. LH reference range: 2.4–12.6 mIU/mL for the follicular phase. E2 reference range: 12.4–233 pg/ml for the follicular phase. PROG reference range: < 0.05–0.893 ng/ml for the follicular phase. PRL reference range: 4.79–23.3 ng/ml. TESTO reference range:8.4–48.1 ng/dl

**Fig. 4:**
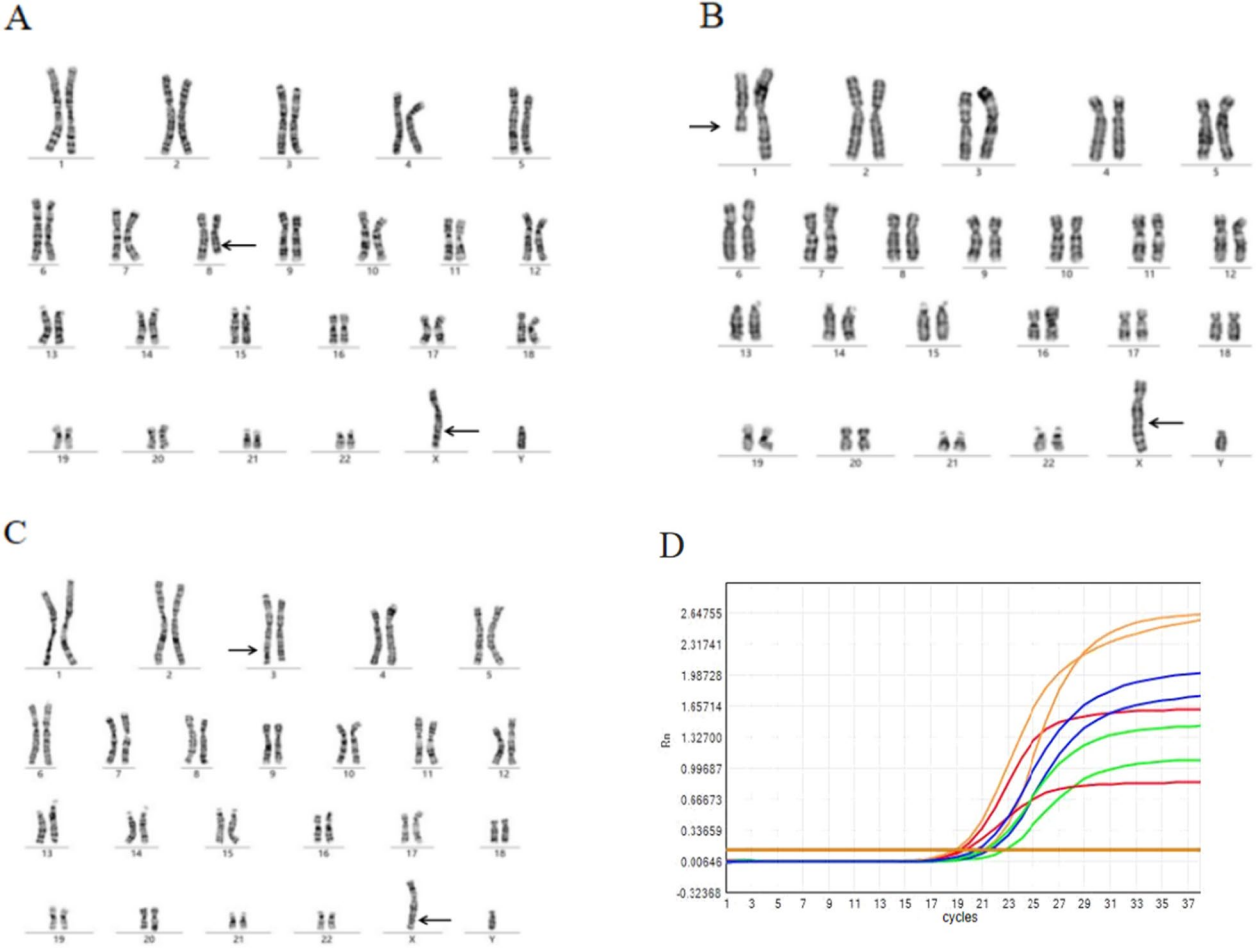
**A** The karyotype was 46,Y,t(X;8)(q26;q22) for case 8. **B** The karyotype was 46,Y,t(X;1)(q26;q23) for case 9. **C** The karyotype was 46,Y,t(X;3)(q26;q24) for case 10. **D** PCR results of the Y chromosome deletions. The AZFa, AZFb and AZFc regions include: sY14 (SRY, blue in tube A), ZFX/ZFY (blue in tube B), sY84 (green in tube A), sY86 (green in tube B), sY127 (orange in tube A), sY134 (orange in tube B), sY254 (red in tube B), and sY255 (red in tube A). Reference value: Typical S-type amplification and curve with Ct < 32

Cases 8, 9 and 10 involved male patients admitted to our hospital for primary infertility. All of these patients underwent karyotype and semen analysis. The karyotypes were detected as 46,Y,t(X;8)(q26;q22), 46,Y,t(X;1)(q26;q23), and 46,Y,t(X;3)(q26;p24) (Fig. 4). The breakpoints were primarily located at Xq26. Multiple semen analyses confirmed the absence of sperm, though endocrine evaluation was normal. Y chromosome microdeletion analysis revealed the presence of a sex-determining region on the Y chromosome and azoospermia factors a, b, and c (AZFa, AZFb, and AZFc) (Table 3).

**Table 3:** Endocrine evaluation, semen analysis and Y chromosome microdeletion test of males with X chromosome translocations

	karyotype	FSH (IU/L)	LH (mIU/mL)	E2 (pg/ml)	PRL (ng/ml)	TESTO(ng/dl)	semen analyse	Y chromosome microdeletions
8	46,Y,t(X;8)(q26;q22)	9.05	11.1	18.7	18.7	451	No sperm	Normal
9	46,Y,t(X;1)(q26;q23)	3.70	2.00	–	10.38	274.94	No sperm	Normal
10	46,Y,t(X;3)(q26;p24)	–	–	–	–	–	No sperm	Normal

Three males with X chromosome translocations underwent endocrine evaluation, semen analysis and Y chromosome microdeletions

FSH reference range: 1.5–12.4 IU/L, LH reference range: 1.7–8.6 mIU/mL, PRL reference range: 4.04–15.2 ng/ml. TESTO reference range:249–836 ng/dl

## Discussion

Human cells possess two sex chromosomes, with males carrying an X and a Y chromosome, and females carrying two X chromosomes. There are more genes on the X chromosome than the Y chromosome. Dosage compensation is achieved by silencing one X chromosome to achieve equal dosages of XX and XY [12]. In early embryogenesis, one of the two X chromosomes in each female embryo cell is randomly inactivated, known as the lyonization [13, 14]. In fact, only partial fractions on the inactivated X chromosome are deactivated and lack transcriptional activity, in other fractions certain gene sites undergo transcription and performed double-dose functions such as primary pseudoautosomal regions (PAR1), secondary pseudoautosomal regions (PAR2), with an X–Y shared fragment of approximately 4 Mb in length located at Xq21.3 [15, 16]. Transcriptional silencing is initiated by an X-chromosome inactivation centre (XIC) located at Xq13. Which X chromosome is inactivated is a random process [17]. If the derived/translocated X chromosome is inactivated, gene silencing spreads to the connected autosomal segment. This phenomenon may cause phenotypic abnormalities [18, 19]. Theoretically, normal phenotypes and gene functions can only be achieved by normal X-chromosome inactivation. Therefore, the difference between which X is inactivated may determine a normal or abnormal phenotype [12].

Here, we report ten cases of X chromosome translocation. An abnormal reproductive system was observed in cases 1, 2 and 3. Extremely low AMH levels indicate ovarian insufficiency. AMH has recently been considered for assessing ovarian reserve, given its high sensitivity and specificity in predicting ovarian response and good intercycle reliability. FSH increases much earlier and more sharply than LH. The FSH/LH ratio is an independent factor to predict poor ovarian response and is associated with poor outcomes in vitro fertilization (IVF) treatment [20]. Patients with the X chromosome translocation and premature ovarian insufficiency (POI) constitute an interesting study in regard to the location of breakpoints. The Xq critical region is known for its role in maintaining ovarian function and normal reproductive lifespan and is located ranging between Xq13 and Xq27 [21–23]. To investigate the effects of balanced X-autosome translocation resulting in POI, Di Battista fine-mapped breakpoints in six patients with POI and balanced X-autosome translocation and addressed gene expression and chromatin accessibility changes in four of them. The results revealed that translocation exerts a broad effect on chromatin structure, this study thus appears to support the hypothesis that positional effects are a causal mechanism for premature ovarian insufficiency associated with X-autosome translocation [24]. The reasonable prevalence of X chromosome structural anomalies and X-autosome translocations related to POI was calculated to range from 4.2 to 12.0% [25].

A significant association between X chromosome translocations and abnormal pregnancy has been documented. X-autosome translocations may occur without a significant detrimental phenotype due to normal X chromosome inactivation, however, the carriers will face increased risks of abnormal pregnancy such as recurrent spontaneous abortion, stillbirths, and congenital disabilities [26]. A female child carrying an unbalanced X-autosome translocation with 45,X,der(X)t(X;14)(q28;q11.2)mat presented poor growth and development. Diagnostic procedures for developmental delay included brain MRI scan showing hypoplasia of the corpus callosum with microcephaly and cerebral atrophy for the derived X chromosome inactivation. Her mother with 46,X,t(X;14)(q28;q11.2) was phenotypically normal [3]. A family with 4 women of 3 different generations carrying an unbalanced X chromosome translocation with 46,X,der(X)t(X;7)(q26;q35) has been reported. None of the carriers showed intellectual disability, and all of them had a very mild clinical presentation mainly characterized by gynaecological/hormonal issues and autoimmune disorders. The XCI pattern showed a skewed X inactivation pattern with a preferential activation of the normal X [27]. Unpredictable detrimental phenotypes associated with skewed XCI patterns may occur in female carriers, whereas azoospermia or severe oligozoospermia occurs in all male carriers [28, 29].

X–Y chromosome translocation is a rare event, and the major breakpoints are located at Xq22 and Yq11 [30–32]. The clinical phenotype is heterogeneously associated with the size of the Xp deletion and the genes involved. For the XCI pattern preferring to the derived X chromosome silencing females with t(X;Y) are generally phenotypically normal except for short stature when the short stature homeobox (SHOX) gene is absent [33]. Patients may have ichthyotic skin disease and Kallmann syndrome when the STS and KAL1 genes are deleted [34]. Intellectual disability is linked to loss of the VCX-A gene [34]. Additionally, other genes have been mapped to Xp22.3 including X-linked recessive chondrodysplasia punctata (CDPX) and ocular albinism type I (OA1) [35]. A familial maternally inherited X–Y chromosome translocation encompassing SHOX and ARSE was documented in two Moroccan siblings with sensorineural deafness. The mother was short in stature but had normal intelligence and no hearing loss [36]. Although this X–Y chromosome translocation was maternally derived, the mother's phenotype was mild and her sons had abnormalities [37–39].

Chromosomal abnormalities are the primary genetic factors that lead to azoospermia and male infertility. Even if the copy number of the cellular chromosome is balanced, almost all hemizygous males with X-autosomal translocations are infertile [40]. Choi reported a 26-year-old male seeking initial infertility evaluation. The detailed physical examination and laboratory tests were normal, with the exception of an abnormal karyotype with a reciprocal translocation at chromosomes X and 16. An open testicular biopsy demonstrated late maturation arrest at the spermatid stage without evidence of significant peritubular fibrosis or hyalinization on pathology, thus confirming significantly reduced reproductive potential [41]. With immature sperm present on testicular biopsy, carriers may be candidates for testicular excisional sperm extraction using intracytoplasmic sperm injection (ICSI) and in vitro fertilization (IVF) [29].

## Conclusions

Our study provides insights into individuals with the X chromosome translocations. Clinical phenotypes are variable and unpredictable with regard to differences in breakpoints and XCI patterns, including phenotypically normal, ovarian insufficiency, infertility, a high risk of birth defects and other syndromes for related genes loss. The effects of different breakpoints and XCI patterns are worthy of further study. Our results suggest that physicians should focus on the characteristics of the X chromosome translocations and provide personalized clinical evaluations in genetic counselling.

## Methods

This study was approved by the Institutional Research Ethics Committee of Jiangxi Maternal and Child Health Hospital. All patients agreed to participate in the study and provided written informed consent. A total of 30,815 patients were referred for karyotyping for genetic counselling at the medical genetic centre between January 2021 and February 2023. Indications for karyotype analysis included infertility, amenorrhea, history of spontaneous abortion, and history of abnormal pregnancy.

### Karyotype analysis

Peripheral lymphocyte blood specimens were subjected to cell culture and harvest, and slides were prepared according to standard operation protocols. Sufficiently dried slides were treated with trypsin at 37 °C and Giemsa staining for G-bands [42]. If necessary, the slides were incubated in Ba(OH)2 at 50 °C for denaturation of chromosomes and then in 2× SSC at 60 °C for reannealing to promote the visualization of highly repetitive C-band regions [43]. Thirty cells were counted, and five cells were analysed by two physicians according to International System for Cytogenetic Nomenclature (ISCN 2020).

### CNV-seq analysis

DNA was extracted from blood or amniotic fluid using a DNeasy Blood and Tissue Kit (Qiagen, Germany) and generated to an average fragment size of 200 bp. All samples passing quality control (> 500 ng; OD260/OD280 > 1.8; OD260/OD230 > 1.5) were prepared for library construction and sequenced using the MGISEQ-2000 platform. By using 100 kb as a basic unit of analysis, all data were aligned to the human reference genome (GRCh37) [44].

### Endocrine evaluation

Blood samples were collected on Days 2–4 of the menstrual cycles and measured using an automatic chemiluminescence immunoassay instrument (Cobas E801, Roche). Levels of FSH, LH, E2, PROG, PRL, T and AMH were quantified according to the instruments of the corresponding kits (Roche, Germany) [20].

### Semen analysis and Y chromosome microdeletions

Two separate semen samples were centrifuged at 3000 × g for 15 min and subjected to microscopic evaluation [45].

Whole blood genomic DNA was extracted using BloodGen Mini Kit (Tegen, Shanghai), and polymerase chain reaction amplification was performed using a PCR instrument (Roche LightCycler 480). Each set of PCRs was carried out via duplex PCR. The primers used included those targeting SRY (sY14), ZFX/ZFY, AZFa (sY84, sY86), AZFb (sY127, sY134), AZFc (sY254, sY255) [46].

## Data Availability

All data generated or analysed during this study are included in the article.

## Abbreviations

FSH: Follicle-stimulating hormone
LH: Luteinizing hormone
E2: Oestradiol
PROG: Progesterone
PRL: Prolactin
T: Testosterone
AMH: Anti-Müllerian hormone
POI: Primary ovarian insufficiency
CNV-seq: Copy number variation sequencing
AZF: Azoospermia factor
PCR: Polymerase chain reaction
